# Endothelin Receptor Antagonists as a Potential Treatment of Diabetic Nephropathy: A Systematic Review

**DOI:** 10.7759/cureus.19325

**Published:** 2021-11-07

**Authors:** Noorain Ahmad, Harish Veerapalli, Chetan Reddy Lankala, Everardo E Castaneda, Afia Aziz, Amy G Rockferry, Pousette Hamid

**Affiliations:** 1 Internal Medicine, California Institute of Behavioral Neurosciences & Psychology, Fairfield, USA; 2 Neurology, California Institute of Behavioral Neurosciences & Psychology, Fairfield, USA

**Keywords:** proteinuria, diabetic nephropathy, avosentan, atrasentan, bosentan, endothelin receptor antagonists, diabetes mellitus

## Abstract

Diabetic nephropathy is becoming a more predominant cause of end-stage renal disease, as the prevalence of diabetes mellitus worldwide is on the rise. In this systematic review, we aimed to define the role of endothelin receptor antagonists, in the prevention and treatment of diabetic nephropathy, in addition to determining their safety. For this review, PubMed, Google Scholar, and Cochrane Library databases, in addition to ClinicalTrials.gov, were searched for publications in the last 20 years. We included 14 studies, seven randomized control trials, and seven post hoc analyses in this paper. Atrasentan decreased albuminuria, reduced blood pressure, and improved lipid profiles with more manageable fluid overload-related adverse events than avosentan and bosentan. Overall, endothelin receptor antagonists, in combination with renin-angiotensin-aldosterone system inhibitors, effectively reduce albuminuria and prevent the progression of diabetic kidney disease. However, more extensive clinical trials still need to be conducted to confirm these relationships and to learn more about the specific factors affecting their efficacy in individual patients.

## Introduction and background

According to the World Health Organization (WHO), approximately 422 million people suffer from diabetes mellitus worldwide, regardless of their economic status [1]. With the rising prevalence of diabetes over the last few decades due to rapid urbanization and progressively sedentary lifestyles, there has also been an increase in its associated complications [2]. Diabetes is among the leading causes of end-stage renal disease (ESRD), with approximately one in three adults with diabetes eventually developing chronic kidney disease [3]. Diabetic nephropathy is characterized by thickening of the glomerular basement membrane, mesangial expansion, and hyaline accumulation in the afferent and efferent arterioles [4]. Eventually, this leads to glomerular hyperfiltration, proteinuria, fall in glomerular filtration rate (GFR) and ESRD [4,5]. Microalbuminuria is one of the earliest detectable clinical indices of renal involvement in diabetes, which is useful in predicting the progression of the disease and the risk of additional cardiovascular mortality [6,7]. Albuminuria is not just a marker of diabetic kidney disease, but it also directly plays a role in the progressive renal damage that occurs [8]. The urinary albumin excretion rate (UAER) is used to clinically categorize diabetic kidney disease into the following stages: normoalbuminuria (UAER < 30 mg/g creatinine), microalbuminuria (UAER 30-300 mg/g) or macroalbuminuria (UAER > 300 mg/g) [7]. The urine albumin creatinine ratio (UACR) is also used in addition to the estimated glomerular filtration rate (eGFR), to classify diabetic kidney disease into three categories: A1 (<3 mg/mmol), A2 (3-30 mg/mmol), and A3 (>30 mg/mmol) [9].

For over two decades, the standard treatment of diabetic nephropathy has focused on strict glycemic control and blood pressure control with angiotensin-converting enzyme (ACE) inhibitors or angiotensin receptor blockers (ARBs) [10]. Although blockade of the renin-angiotensin-aldosterone system (RAAS) reduces the degree of proteinuria, ACE inhibitors and ARBs are not efficient in preventing disease progression, and they may even increase the long-term risk for ESRD [11,12]. They tend to exhibit a phenomenon of "late escape," which is the recurrence of proteinuria even with RAAS blockade [10]. Other potential treatments for diabetic nephropathy include mineralocorticoid receptor antagonists, phosphodiesterase inhibitors (pentoxifylline), pyridoxamine, apoptosis signal-regulating kinase 1 (ASK1) inhibitors, bardoxolone methyl, and endothelin (ET) receptor antagonists [13]. However, such treatments remain controversial, and thus, additional research and clinical evidence are required to determine their efficacies.

Endothelin (ET) receptor antagonists are a promising group of drugs for reducing albuminuria in patients with diabetic kidney disease. In fact, there are no published studies of a medication added to RAAS antagonists that reduces albuminuria as effectively as ET receptor blockers [14]. There are substantial clinical evidence for increased plasma endothelin-1 (ET-1) levels in patients with diabetes mellitus, leading to endothelial dysfunction [12]. The endothelin system consists of three isoforms, ET-1, ET-2, and ET-3. The former is the principal isoform present in the kidney and is present in glomerular cells, renal endothelial cells, and renal tubules [15,16]. ET-1 is a potent vasoconstrictor peptide that plays a pivotal role in controlling cell proliferation and regulating the accumulation of extracellular matrix and inflammatory cells, leading to fibrosis [16]. The ETA and ETB receptors are the primary G-protein coupled receptors by which ET-1 carries out its actions [15]. ETA receptors are found primarily in vascular smooth muscle cells; their activation mediates vasoconstriction, insulin resistance, inflammation, and fibrosis, which causes endothelial dysfunction. ETB receptors are present in vascular endothelial cells and have anti-proliferative effects. Activation of ETB receptors causes vasodilation through nitric oxide or prostacyclin production [12].

There has been plenty of research identifying the role of ET receptor antagonists in the prevention of diabetic nephropathy-associated albuminuria in experimental studies. More recently, several randomized clinical trials (RCTs) have been conducted to ascertain the role of these drugs in humans. ETA selective receptor antagonists prevent inflammatory and cytoskeletal changes in podocytes induced by ET-1. ETB receptor blockade is associated with higher adverse effects such as sodium and water retention and decreased nitric oxide production. Therefore, more clinical trials have focused on ETA selective blockade with atrasentan (ETA: ETB blockade ~1200:1) or avosentan (ETA: ETB blockade ~50-300:1), rather than non-selective blockade with bosentan (ETA: ETB blockade ~20:1) [17]. However, the use of ETA receptor antagonists remains controversial, as increasing doses of even highly selective antagonists can still lead to adverse effects of fluid retention and heart failure. Further research is still required to establish the definitive role of ET receptor antagonists. This systematic review of available clinical data from RCTs on ET receptor antagonists aimed to determine their therapeutic role and safety in preventing diabetic nephropathy and ESRD.

## Review

Methods

The Preferred Reporting Items for Systematic Reviews and Meta-Analyses (PRISMA) statement [18] was used to perform this systematic literature review.

Search Strategy:

A comprehensive data search was performed using the databases PubMed, Google Scholar, and Cochrane Library. The databases were searched for papers published in the last two decades, from January 1, 2001, to June 20, 2021. Medical Subject Heading (MeSH) terms and the following keywords were searched in various combinations: "Diabetes mellitus," "Endothelin receptor antagonists," Bosentan, Avosentan, Atrasentan, "Diabetic nephropathy," and proteinuria. In addition, ClinicalTrials.gov was accessed to retrieve relevant clinical trials. 

Study Screening and Selection:

The titles and abstracts of the search results were screened for relevancy of information after the removal of duplicate articles. The remaining publications were then individually screened by two reviewers, using the following inclusion criteria: (1) Studies published between 2001-2021 (2) Studies published in the English language (3) Human studies (4) Randomized control trials comparing endothelin receptor antagonists with placebo (5) Studies performed in diagnosed diabetic patients (aged ≥18 years), with evidence of proteinuria and decline in glomerular filtration rate Studies in languages other than English, animal research studies, studies involving non-diabetic patients, and studies that were not randomized control trials were excluded. 

Data Extraction and Quality Assessment:

Two researchers independently evaluated the whole text of the remaining publications to extract data. The author names, year of publication, study design, sample size, interventional drugs, and study outcomes were noted. The same reviewers then assessed the quality of the final eligible studies using the Cochrane risk of a bias tool for RCTs [19]. Selection bias, performance bias, detection bias, attrition bias, reporting bias, and other prejudice are all covered by this assessment tool. Assessments were made for one or more items within each domain, covering several features of the domain or distinct outcomes. For each item, the tool classified material bias risk as either high, low, or unclear [19]. Any differences were resolved by a third author's assessment.

Results

**Figure 1:**
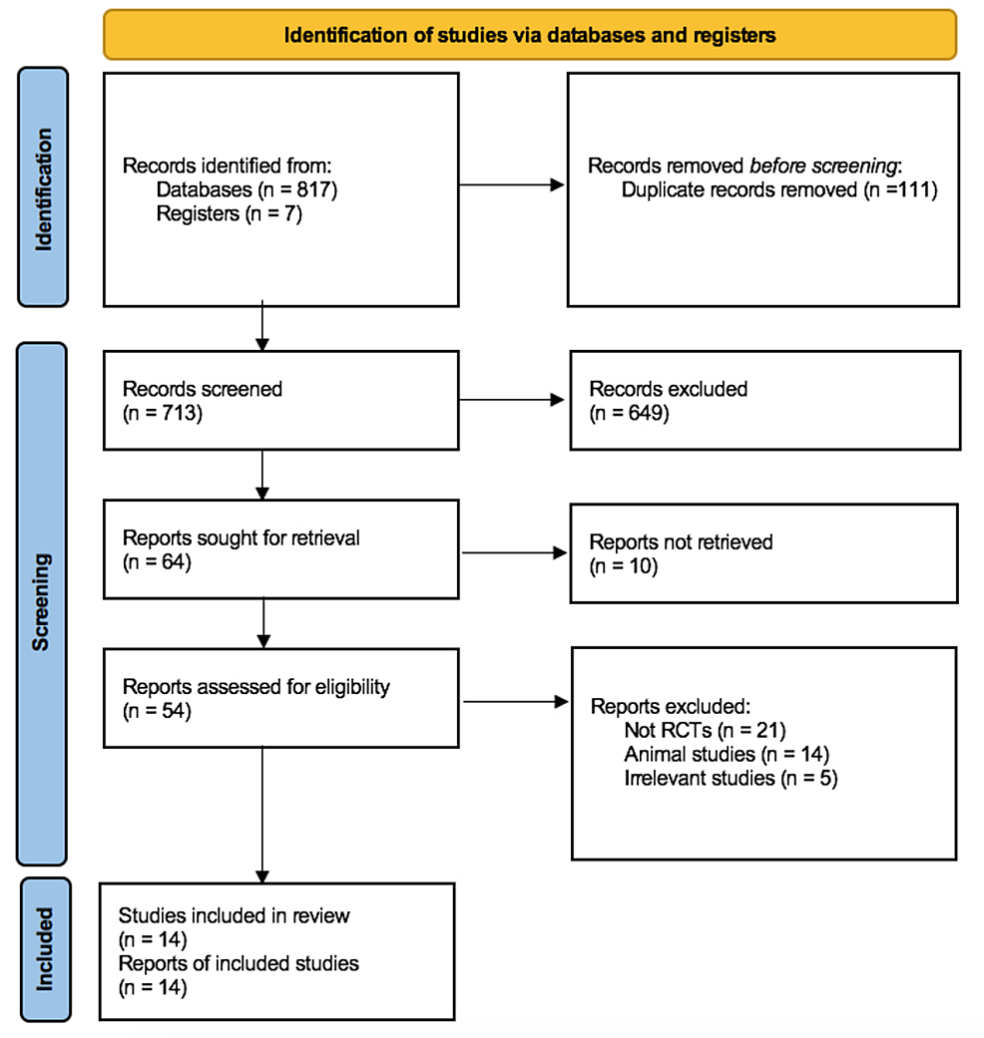
PRISMA Flow Diagram PRISMA: preferred reporting items for systematic reviews and meta-analyses

Literature Search:

A total of 824 articles were initially identified through our search of PubMed, Google Scholar, Cochrane Library, and ClinicalTrials.gov. After removing duplicate studies and screening the titles and abstracts, 54 articles remained. Eventually, 14 studies were included in this systematic review, of which seven are post hoc analyses. Figure 1 depicts the sequence of study identification and subsequent inclusion.

Characteristics of the Included Studies:

The following 14 studies were included in this review: Wenzel et al. [20], Mann et al. [8], Rafnsson et al. [21], Kohan et al. [22], Andress et al. [23], de Zeeuw et al. [14], Kohan et al. [24], Schievink et al. [25], Pena et al. [26], Koomen et al. [27], Lin et al. [28], Webb et al. [29], Heerspink et al. [30], Koomen et al. [31]. Of the selected articles, 11 studied atrasentan [14,22-31], two avosentan [8,20], and one bosentan [21].

All RCTs evaluated the efficacy and adverse effects of various doses of the interventional drug versus placebo. The participants of all studies were diagnosed as people with diabetes showing clinical evidence of albuminuria and were already on stable doses of ACE inhibitors or ARBs. However, in the study on bosentan, 19 out of 24 participants and 20 of 22 participants in the interventional and control groups, respectively, were taking RAAS inhibitors [21]. The efficacy outcomes of relevance were changes in albuminuria (measured by UACR or UAER), eGFR, weight, blood pressure, hemoglobin, and lipid profiles. Doubling of serum creatinine, progression to end-stage renal disease and occurrence of edema events were used as safety markers. The characteristics of the included studies are presented in Table 1.

**Table 1:** Table of Features and Outcomes of Studies Included in the Systematic Review

Avosentan	Author & Year of Publication	Study Design	Associated NCT Number	Number of Patients	Interventional Drug Doses	Purpose of the Study	Outcomes	Adverse Events	Conclusion
	Wenzel et al. [20], 2009	Randomized, double-blind, placebo-controlled trial	N/A	286	5, 10, 25, 50 mg/day or placebo	To determine the effect of avosentan on urinary albumin excretion rate (UAER) in patients with diabetic nephropathy.	Mean UAER levels at baseline ranged from 0.79 ± 0.79 mg/min in the 10 mg group to 1.21 ± 1.43 mg/min in the 50 mg group.	161 patients (56.3%) reported adverse events, most of which were mild to moderate in severity.	Avosentan given in addition to standard treatment decreases UAER in patients with diabetic macroalbuminuria. The incidence of adverse effects was significantly elevated, especially with high dosages of avosentan. Avosentan dosages over 25 mg appear to have no extra antiproteinuric effect; hence, the ideal dosage in terms of risk-benefit ratio may be defined as 10 mg.
							Relative to baseline, UAER decreased significantly with avosentan 5, 10, 25, and 50 mg, respectively (−20.9, −16.3, −25.0, and −29.9%) but increased with placebo (35.5%).	21 (7.3%) patients experienced adverse events that led to withdrawal from study medication.	
							Avosentan 5, 10, 25, and 50 mg decreased median relative UAER levels by −28.7, −42.2, −44.8, and −40.2%, respectively, versus a 12.1% increase with placebo.	The main adverse events were peripheral edema (12%), mainly with high (≥25 mg) dosages of avosentan.	
							Total cholesterol decreased 7 to 17 mg/dl with avosentan and was increased in placebo.		
	Mann et al. [8], 2010	Randomized, double-blind, placebo-controlled trial	NCT00120328 (ASCEND)	1392	25, 50 mg/day or placebo	To examine the effect of avosentan on time to doubling of serum creatinine, ESRD, or death. In addition, changes in urine albumin excretion, eGFR, and cardiovascular outcomes were also evaluated.	Avosentan significantly reduced UACR in patients who were treated with avosentan 25 mg, 50 mg, and placebo. The median reduction in UACR was 44.3, 49.3, and 9.7%, respectively.	Avosentan had substantially higher adverse events than placebo (19.6 and 18.2 percent against 11.5 percent).	Avosentan reduces albuminuria but induces significant fluid overload and congestive heart failure. The trial was terminated early due to an excess of cardiovascular events. There was no detected difference in the frequency of the primary outcome between groups.
							The eGFR declined in all three groups by 2.5 to 4 ml/min per 1.73 m^2^ during six months.	Death occurred in 21 (4.6%), 17 (3.6%), and 12 (2.6%), respectively.	
							BP declined by 0.0 to -0.5 mmHg systolic and diastolic with placebo and by −34.1 to −6.1 mmHg systolic and −3.0 to −4.4 mmHg diastolic in both avosentan groups.	Mean ± SD hemoglobin levels decreased in patients who were taking avosentan 25 mg by 11.4 ± 11.7 g/L, avosentan 50 mg by 11.0 ± 12.6 g/L, and placebo by 0.1 ± 9.0 g/L from baseline.	
								Mean ± SD body weight increased by 0.4 ± 3.0, 0.3 ± 2.9, and 0.0 ± 2.7 kg, respectively at 3 months.	
Bosentan	Author & Year of Publication	Study Design	Associated NCT Number	Number of Patients	Interventional Drug Doses	Purpose of the Study	Outcomes	Adverse Events	Conclusion
	Rafnsson et al. [21], 2012	Randomized, double-blind, placebo-controlled trial	NCT01357109 (BANDY)	56	250 mg/day or placebo	To test if bosentan improves peripheral endothelial function by looking at changes in the reactive hyperemia index (RHI) and flow-mediated dilatation in the brachial artery (FMD).	RHI increased from 1.73 ± 0.43 at baseline to 2.08 ± 0.59 in the bosentan group but did not change in the placebo group.	Three patients withdrew because of adverse events.	Bosentan improved peripheral endothelium-dependent vasodilatation, whereas no change was observed in brachial artery FMD.
							There was no significant change in UACR.	Bosentan treatment resulted in a drop in hemoglobin from 134 to 127 g.	
							Brachial artery FMD and blood pressure did not change during treatment.		
							Changes in lipid profile or blood glucose levels were non-significant.		
Atrasentan	Author & Year of Publication	Study Design	Associated NCT Number	Number of Patients	Interventional Drug Doses	Purpose of the Study	Outcomes	Adverse Events	Conclusion
	Kohan et al. [22], 2011	Randomized, double-blind, placebo-controlled trial	N/A	89	0.25, 0.75, 1.75 mg/day or placebo	The primary outcome was to compare each treatment group's weekly change in UACR from baseline to placebo.	In the placebo group, 17% of subjects achieved >40% reduction in UACR from baseline compared with 30, 50, and 38% in the 0.25, 0.75, and 1.75 mg groups.	Peripheral edema occurred in 9% of placebo-treated participants and 14, 18, and 46% of 0.25, 0.5, and 1.75 mg atrasentan-treated subjects.	The impact of the 0.75 and 1.75 mg dosages on UACR was maintained, but not in the 0.25 dose group. Although both effective dosages were linked to a substantial drop in blood pressure, the main effect of atrasentan on UACR reduction was independent of blood pressure changes.
							The mean change of systolic BP was −0.3 mmHg (vs. placebo) in the 0.25 mg group, −8.8 mmHg in the 0.75 mg group, and −7.6 mmHg in the 1.75 mg group.	One patient discontinued due to serious fluid retention-related adverse events (patient had high baseline BNP levels).	
							The mean of diastolic BP was −0.5 mmHg in the 0.25 mg group, −5.8 mmHg in the 0.75 mg group, and −7.4 mmHg in the 1.75 mg group.		
							Compared to 0.1 g/dl for placebo, hemoglobin change was −0.7 g/dl in the 0.25 mg group, −0.4 g/dl in the 0.75 mg group, and −0.9 g/dl in the 1.75 mg group.		
	Andress et al. [23], 2012	Post hoc analysis of a randomized, double-blind, placebo-controlled trial (Kohan et al., 2011)	N/A	89	0.25, 0.75, 1.75 mg/day or placebo	To further characterize the edema events that occurred, to determine changes in biomarkers, to assess differential factors for efficacy and safety among different ethnicities, and compare the response in patients receiving maximum RAAS inhibitors with others.	UACR was decreased in the 0.75 mg and 1.75 mg groups.	Edema was reported in 21 subjects.	Edema occurrence with atrasentan was dose-dependent, mostly mild or moderate. The finding that NT-pro-BNP levels were not increased with atrasentan exposure is consistent with the clinical safety profile of low-dose treatment in high-risk populations. Patients receiving maximum RAS inhibitor doses had a similar beneficial response to those patients receiving less than maximal RAS inhibition.
							Mean UACR reduction in those taking the maximum doses of RAAS inhibitors was 32% in the 0.75 and 35% in the 1.75 mg groups.	The incidence of mild or moderate edema was: 2/23, 4/22, 5/22, and 10/22 for placebo, 0.25mg, 0.75 mg, and 1.75 mg, respectively; none reported severe edema.	
							Changes in serum IL-6, NT-pro-BNP, ET-1, urine TGFb, or MCP-1 were not significant.		
							Urinary neutrophil gelatinase-associated lipocalin (NGAL) was reduced 24% in the 1.75 mg group.		
							Hispanic subjects (58%) tended to have more significant UACR reductions than non-Hispanics without different rates of edema.		
	de Zeeuw et al. [14], 2014	Two identically designed, parallel, multinational, randomized, double-blind, placebo-controlled phase IIb trials	NCT01356849 (RADAR) NCT01424319 (JAPAN)	211	0.75, 1.25 mg/day or placebo	To examine the balance between albuminuria-lowering benefits and fluid retention side effects and to test the effectiveness and safety of atrasentan on albuminuria and other renal risk-related measures.	UACR ratios were decreased by an average of 35% and 38% in the 0.75 mg and 1.25 mg groups, respectively.	Although there were no differences in the rates of peripheral edema and heart failure across groups, more patients treated with 1.25 mg/d withdrew owing to side events.	In conclusion, atrasentan reduced albuminuria, improved BP and lipid spectrum with manageable fluid overload–related side effects.
							0.75 mg and 1.25 mg groups reduced albuminuria ≥30% in 51% and 55% of participants, respectively.	The use of atrasentan was associated with a significant increase in weight and a reduction in hemoglobin.	
							eGFR and office BP measurements were unchanged.		
							24-hour systolic and diastolic BP, LDL cholesterol, and triglyceride levels decreased significantly in both treatment groups.		
	Kohan et al. [24], 2015	Post hoc analysis of two randomized, double-blind, placebo-controlled, phase IIb trials (RADAR/JAPAN)	NCT01356849 (RADAR) NCT01424319 (JAPAN)	211	0.75, 1.25 mg/day or placebo	To determine the baseline parameters that predict atrasentan-associated fluid retention, using weight gain and hemoglobin (Hb) as proxies for fluid retention. Another aim was to determine if the degree of fluid retention necessarily correlated with the magnitude of albuminuria reduction.	Predictors of weight gain within two weeks of treatment were: higher atrasentan dose, lower eGFR, higher glycated hemoglobin, increased systolic BP, and lower homeostatic metabolic assessment product.	Bodyweight increased by approximately 1 kg after two weeks of treatment compared with a decrease of 1 kg in the control group.	Fluid retention was more likely in patients who had lower eGFR or received a higher dose of atrasentan. Albuminuria reduction was not related to changes in weight and Hb.
							Baseline predictors of Hb change were atrasentan dose 0.75 or 1.25 mg/d versus placebo and lower eGFR.	Hb decreased by 1 g/dl in both atrasentan groups after two weeks of treatment.	
							There was no difference between UACR responders and non-responders in changes in body weight or Hb.		
	Schievink et al. [25], 2015	Post hoc analysis of two randomized, double-blind, placebo-controlled, phase IIb trials (RADAR/JAPAN)	NCT01356849 (RADAR) NCT01424319 (JAPAN)	164 patients who had a complete risk marker profile at baseline and follow-up	0.75, 1.25 mg/day or placebo	To use the Parameter Response Efficacy (PRE) score to predict the effect of atrasentan on renal and heart failure outcomes.	The PRE score was used to predict renal risk changes of −23% for 0.75mg atrasentan and −30% for 1.25 mg.	PRE scores predicted a small non-significant increase in heart failure risk for atrasentan 0.75 and 1.25 mg/day (+2% vs. +7%).	Based on short-term variations in risk markers, both atrasentan 0.75 and atrasentan 1.25 mg/day are expected to decrease renal risk and slightly increase heart failure risk, the latter to a lesser extent with the low dose.
							By limiting the population to responders (>30% albuminuria reduction), there was a mean decrease in albuminuria (60%) for the 0.75 mg/day and 1.25 mg/day dose. Non-responders had no significant change in albuminuria.		
	Pena et al. [26], 2017	Post hoc analysis of a randomized, double-blind, placebo-controlled, phase IIb trial (RADAR)	NCT01356849 (RADAR)	150	0.75, 1.25 mg/day or placebo	To assess the effect of atrasentan on a pre-specified panel of 13 urinary metabolites known to reflect mitochondrial function, using urine samples collected during the RADAR study.	At baseline, only nine of the 13 urinary metabolites were detectable in urine.	N/A	In conclusion, urinary metabolites linked to mitochondrial function were stabilized with atrasentan 1.25mg/d, but not between placebo or 0.75mg/d. Changes in individual metabolites and the metabolite index correlated with changes in eGFR over time but did not correlate with changes in UACR.
							In patients with baseline eGFR <60ml/min/1.73m^2 ^treated with placebo, concentrations of the metabolites decreased during 12-weeks follow-up. In contrast, the same metabolites remained stable in those receiving atrasentan.		
							Relative to placebo, seven of the nine metabolites were increased in atrasentan 0.75mg/d, and all nine metabolites were increased in atrasentan 1.25mg/d.		
							In patients treated with 1.25mg/d, all individual metabolites remained increased relative to placebo 30-days after stopping treatment.		
	Koomen et al. [27], 2018	Post hoc analysis of two randomized, double-blind, placebo-controlled, phase IIb trials (RADAR/JAPAN)	NCT01356849 (RADAR) NCT01424319 (JAPAN)	161 (Only data from the atrasentan receiving groups was studied)	0.75 or 1.25 mg/day	To identify the optimal dose of atrasentan with maximal albuminuria reduction and minimal signs of sodium retention, as manifested by an increase in body weight.	UACR decreased by 34.0% and 40.1%, respectively, in the 0.75 and 1.25 mg groups.	The mean increase in body weight with 0.75 and 1.25 mg of atrasentan was 0.9 kg and 1.1 kg, respectively.	The observed variation in albuminuria and bodyweight response correlated to the variation in the estimated individual pharmacokinetic parameters of atrasentan. At the atrasentan, C_trough_ equivalent to the administration of 0.75 mg of atrasentan, a clinically relevant reduction in albuminuria was observed with fewer signs of sodium retention in comparison to a C_trough_ equivalent to the administration of 1.25 mg of atrasentan.
							The exposure‐response curves for albuminuria and weight crossed at a mean C_trough_ of approximately 0.75 mg of atrasentan per day.	At the mean C_trough_ of the 1.25 mg dose, a greater albuminuria response was observed at the expense of a larger increase in bodyweight.	
	Lin et al. [28], 2018	Post hoc analysis of three randomized, double-blind, placebo-controlled, phase II trials	NCT01356849 (RADAR) NCT01399580 NCT01424319 (JAPAN)	257	Studies 1 and 3: 0.75, 1.25 mg/day or placebo Study 2: 0.5, 1.25 mg/day or placebo	To describe the pharmacokinetic characteristics of atrasentan as well as the exposure-response correlations for UACR and the adverse events (peripheral edema). Potential differences in Western patients compared to Japanese patients were investigated.	Population pharmacokinetic analysis–predicted results suggested that atrasentan doses of 0.5, 0.75, and 1.25 mg/d would achieve mean atrasentan concentrations of 0.92, 1.9, and 3.4 ng/ mL, respectively, which correspond to estimated median UACR reductions of 31%, 37%, and 41 %, respectively.	No statistically significant exposure-edema relationship was identified in the current regression analysis at the doses of 0.5, 0.75, and 1.25 mg, the rate of peripheral edema appeared to increase slightly with an increase in atrasentan exposure.	Between Western and Japanese patients, the exposure-response correlations for effectiveness and tolerability were consistent. Based on these findings, a dosage of 0.75 mg/d was chosen for the next Phase III (SONAR) study.
	Webb et al. [29], 2017	Randomized, double-blind, placebo-controlled, phase IIb trial	N/A	48	0.5, 1.25 mg/day or placebo	To see if atrasentan increased thoracic fluid accumulation (lowered thoracic bioimpedance) and if bioimpedance changes were related to changes in weight, peripheral edema, or diuretic use.	The placebo group had minor fluctuations of bioimpedance from baseline (0.5–1.1 Ohms).	Peripheral edema rates were increased in all categories (1.25-mg group showing the largest increase).	Between the treatment groups and placebo, there were no significant variations in bioimpedance. Atrasentan caused weight gain and peripheral edema while lowering albuminuria and hemoglobin.
							The atrasentan groups showed mean reductions of 1.7 and 2.0 Ohms with (0.5 mg dose and 1.25 mg dose, respectively, amounting to nadir mean declines of 7 and 11% from baseline.	The atrasentan 0.5 mg and 1.25 mg groups saw overall substantial weight increases of 1.7 and 1.6 kg, respectively.	
							At weeks 2 and 4, both atrasentan groups exhibited increases in bioimpedance of 16 and 21%, respectively, from their nadir.	The 0.5 mg group had a 0.47 g/dl drop, while the 1.25 mg group had a 0.84 g/dl decrease in hemoglobin.	
	Heerspink et al. [30], 2019	Randomized, double-blind, placebo-controlled, phase III trial	NCT01858532 (SONAR)	2648	0.75 mg/day or placebo	To assess the efficacy of atrasentan in delaying the progression of CKD	UACR decreased by 51.8% from baseline during the enrichment period. UACR increased more in the placebo group during the double-blind treatment period.	A composite renal endpoint event occurred in 79 (60% ) participants in the atrasentan group and 105 (79% ) in placebo.	When compared to placebo, low-dose atrasentan treatment followed by long-term therapy significantly reduced the risk of the main composite renal outcome. However, in responders, hospitalization for heart failure was greater with atrasentan than with placebo, emphasizing the need for continuing close monitoring of these side events.
							The mean rate of change in eGFR in the atrasentan group was -2.4 mL/min per 1.73 m^2^ and -3.1 mL/min in the placebo group.	Fluid retention and anemia were more common in the atrasentan group.	
							BP dropped by 6.1 mmHg during the enrichment period, but only -1.6 mmHg following randomization.	In the atrasentan group, 47 (35% ) patients were admitted to the hospital for heart failure, compared to 34 (26% ) patients in the placebo group.	
							The mean difference in body weight was 0.2kg, and the increase in BNP from randomization was 10.5% greater with atrasentan than placebo.		
	Koomen et al. [31], 2020	Post hoc analysis of the enrichment period of the SONAR trial (a randomized, double-blind, placebo-controlled, phase III trial)	NCT01858532 (SONAR)	4775	0.75 mg/day during the 6-week enrichment period of the SONAR trial	To determine whether atrasentan exposure explains between-patient variability in UACR response (a substitute for kidney protection) and B-type natriuretic peptide (BNP) response (a proxy for fluid retention). The area under the plasma concentration-time curve (AUC) was calculated using clearance (CL) and volume of distribution (Vd).	Median UACR change at the end of the enrichment period was −36.0%, and median BNP change was 8.7%, which varied. Higher atrasentan AUC was associated with greater UACR reduction and greater BNP increase independent of eGFR, hemoglobin, or BNP.	N/A	Between-patient variability in efficacy and safety of 0.75 mg atrasentan could be attributed in part to atrasentan plasma exposure and patient characteristics.

**Figure 2:**
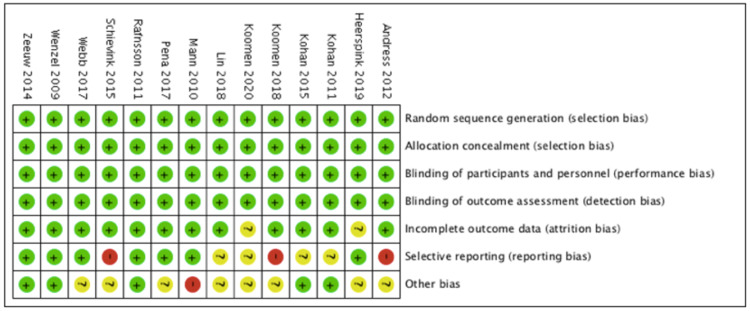
Quality Assessment Graph Green indicates a low risk of bias, red indicates a high risk of bias, and yellow indicates an unclear risk of bias. The following studies were reviewed for quality assessment: Andress et al. [23], Heerspink et al. [30], Kohan et al. [22], Kohan et al. [24], Koomen et al. [27], Koomen et al. [31], Lin et al. [28], Mann et al. [8], Pena et al. [26], Rafnsson et al. [21], Schievink et al. [25], Webb et al. [29], Wenzel et al. [20], de Zeeuw et al. [14].

**Figure 3:**
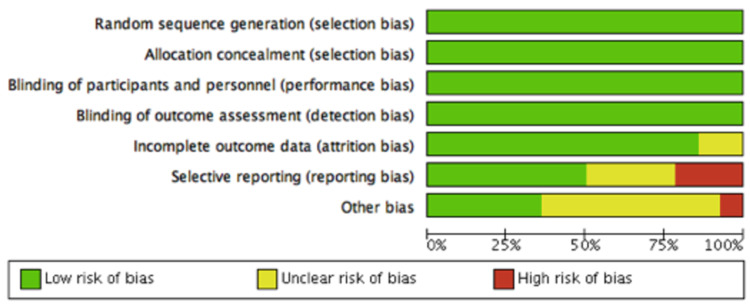
Summary of the Quality Assessment of the Included Studies

Risk of Bias Assessment:

Figures 2, 3 demonstrate the risk of bias assessment of the included studies. Most studies had moderate to low risk of bias, using the Cochrane risk of bias tool [16]. The study Mann et al. [8] was terminated early due to an excess occurrence of cardiovascular outcomes and therefore had a higher risk of bias. 

Discussion

Based on the available clinical data on the use of endothelin receptor antagonists, it is evident that these agents are a promising new hope for the prevention and treatment of diabetic-related kidney disease. The more ETA-selective receptor agents, atrasentan and avosentan, have shown more significant results in reducing albuminuria in high-risk diabetic individuals than bosentan, a dual ETA-ETB receptor antagonist. ETA receptor blockade leads to reduced glomerular vasodilation, altering the glomerular permeability for albumin and thus, lowers the tubular load of albumin [14]. This, in turn, reduces endothelin's inflammatory effects of fibrosis and collagen deposition. In addition, atrasentan and avosentan have also shown assuring results in lowering blood pressure and improving lipid profiles.

However, the ET receptor antagonists are not without adverse effects. There is an indication of a higher risk of cardiovascular and edema-related events occurring with these drugs than placebo. The most commonly reported side effects are edema, hypervolemia, hypotension, anemia, dyspnea, hypoglycemia, and headache [12,32]. Cardiovascular events occurred in four trials [8,14,20,22], and they were most often coronary artery disease, non-fatal acute myocardial infarction, stroke, or congestive heart failure (CHF). Avosentan was associated with a significantly increased risk of CHF compared to atrasentan, which is attributable to the lower selectivity of avosentan for the ETA receptor.

Edema was reported with all three drugs, but there was no significant difference when treatment groups were compared to placebo in a meta-analysis [32]. These findings were also supported by the meta-analysis done by Zhang et al. [12], which found no significant difference in the occurrence of moderate adverse events between the treatment and control groups, but a higher incidence of more severe events in the interventional group was found. It should be noted that edema had a dose-dependent occurrence, as the risk of edema was increased with those treated with a 1.25mg or higher dose of atrasentan [17].

An important observation that was drawn from past clinical studies is that the dose-response associated with fluid retention is different from the dose for the albuminuria-lowering effect [14]. One possibility is that fluid retention is driven by the ETB receptor blocking capacity of the drugs, although there are also reports of sodium retention induced by ETA receptor blockade [14]. Schievink et al. [25] used the parameter response efficacy (PRE) score (an algorithm developed to translate short-term drug effects into predictions of long-term effects on clinical outcomes) to confirm that atrasentan decreases renal risk but slightly increases heart failure risk in a dose-dependent manner. According to many trials and post hoc studies, 0.75 mg/d of atrasentan as an adjuvant to RAAS inhibition is the optimal dose for renal protection with maximal albuminuria reduction and minimum indications of salt retention [14,27].

Bosentan:

Although bosentan did not show significant results of decreasing UACR, Rafnsson et al. [21] found that it could improve endothelial function in diabetic patients with microalbuminuria. This trial had a small number of participants. However, it was an essential step in testing whether an ET receptor antagonist could prevent the micro and macrovascular complications of diabetes mellitus.

Digital endothelial function was measured via pulse amplitude tonometry to determine the change from the baseline of the reactive hyperemia index (RHI). Flow-mediated vasodilation (FMD) of the brachial artery was also determined to assess macrovascular function. RHI was increased in the bosentan group, but the brachial artery FMD did not change, indicating that bosentan improves endothelial function in only small vessels and therefore plays a role in preventing the microvascular changes of diabetes mellitus. As nitroglycerine-induced digital hyperemia was not affected, it was concluded that bosentan did not play a role in endothelium-independent vasodilation [21].

An interesting finding was that plasma ET-1 levels were mildly increased in the treatment group compared to placebo when they would be expected to decrease with an ET receptor antagonist. This phenomenon could be attributed to the fact that the ETB receptor is responsible for the clearance of ET-1. This theory was supported by Andress et al.'s [23] trial findings, in which ET-1 levels did not change due to less effect on the ETB receptor by atrasentan.

The adverse effects of bosentan could not be fully appreciated in this small, short-term trial. Three patients withdrew from the trial due to adverse effects, and only one of them had edema. Since there have not been enough clinical trials studying bosentan, it is hard to determine its safety. However, its possible role in preventing microvascular changes should be further studied in more extensive trials. A significant drop in hemoglobin was found, as seen with avosentan and atrasentan.

Avosentan:

Wenzel et al.'s [20] trial was the first to test the efficacy of avosentan, displaying good results in the albuminuria decreasing capability of avosentan. All doses decreased UAER levels in a dose-dependent manner. Interestingly, the proportion of patients with adverse effects was higher with placebo than avosentan for the lower doses [20]. However, with the higher dose of 50mg, more adverse effects were reported, such as edema, anemia, headache [20]. This dose-dependent relationship showed that adverse events were more likely with higher doses and helped determine the safe dose of avosentan. However, statistical analysis showed that the mortality rates of treatment and control groups were similar [20]. 

Even though Wenzel et al. [20] discovered no more significant benefit of albuminuria reduction beyond the 25mg dose, the Mann et al. [8] trial still tested the 25mg and 50mg doses. These higher doses significantly reduced UACR by 40% to 50% but at the price of severe adverse effects such as fluid overload and CHF and, therefore, higher mortality rates [8]. The trial was terminated early due to the excess of CVS events, which were the most common cause of death (74%). For this reason, the proportion of patients who met the primary composite endpoints was not different among treatment and control groups. The most common reason for patients withdrawing was fluid overload, reported by 44/89, 38/87, and 8/53 participants from the 25mg, 50mg, and placebo groups, respectively [8].

It is believed that avosentan becomes less selective for the ETA receptor at higher doses and leads to sodium and fluid retention effects of ETB receptor blockade, which also explains the slight increase in body weight. Whether the edema-related adverse effects were due to high dosages or to the participants having stage III/IV, CKD is still uncertain requires more research to ascertain the safety of avosentan [8].

The role of avosentan in affecting GFR is unclear as the GFR declined in both avosentan treatment groups as well as the control group. However, there was a more considerable decrease in GFR reported in the 50mg group, which could be attributed to drug-induced fall in intra-glomerular pressure. Although ERSD occurred less frequently with avosentan, it could have been due to early termination of the trial and incomplete results. Overall, mild blood pressure reduction was noted, and lipid profiles were improved with avosentan administration [8,20]. High levels of triglycerides and lower levels of high-density lipoproteins are significantly associated with increased albuminuria in hypertensive women [33]. This association of lipids and albuminuria may explain the effect of ET receptor antagonists on lipid profiles. Non-dose-dependent mild decreases in hematocrit and hemoglobin levels were seen [8,20]. They could have been due to hemodilution or due to the direct effect of diabetic nephropathy. Some studies have also suggested that ACE inhibitors may suppress erythropoiesis [34].

Atrasentan:

More clinical research is available on atrasentan, perhaps due to its higher selectivity for the ETA receptor and its decreased risk of adverse effects at moderate doses. Zhou et al. [17] found that avosentan reduced GFR while atrasentan prevented eGFR decline.

According to Kohan et al. [22], GFR did not significantly change during treatment with atrasentan, suggesting that ET receptor antagonism of efferent vasoconstriction may not be the principal method for its albuminuria lowering effect, unlike the action of RAAS inhibitors. Subgroup analysis of different doses revealed that the higher dosage groups displayed greater GFR reduction than the moderate dosage and control groups, which exhibited lesser eGFR reduction and were able to prevent loss of eGFR more [12]. There were significant reductions in BP compared to placebo, which supports evidence that these drugs can also be useful for preventing hypertension [14,22].

Webb et al. [29] conducted a trial to assess the relationship between atrasentan-associated peripheral edema/weight gain and thoracic bioimpedance. The overall change in bioimpedance values was not significantly different from the placebo. Early decreases in thoracic bioimpedance were found in the treatment group. However, the values started to increase thereafter, most likely because initial occurrences of edema causing the decrease in bioimpedance triggered an increase in endogenous BNP production, which served as a diuretic [29]. It should also be noted that the patients in this study were already taking stable doses of diuretics, which might have prevented severe adverse effects of fluid retention from occurring. The finding that albuminuria reduction was not associated with changes in body weight, hemoglobin, and BNP response suggests that the albuminuria-reducing efficacy of atrasentan is not impaired by fluid retention [24,23,31].

Hemoglobin was significantly reduced in the ET receptor antagonist group (atrasentan and avosentan), as compared to the control, and there was an increased risk of anemia for the treatment group [14,24,29]. The cause of anemia is most likely due to hemodilution because of fluid retention caused by inhibition of the ETB receptor.

Factors Affecting ET Receptor Antagonist Response:

Overall, Caucasian patients had greater albuminuria lowering effect than Black patients [31]. The pharmacokinetics and exposure-response correlations in Japanese and Western patients were estimated to be similar in the post hoc analysis by Lin et al. [28]. However, it was noted that plasma concentrations of atrasentan were higher in the Asian population and the number of Asian participants significantly correlated with the UACR reducing effect [17]. Hispanic patients had a more significant reduction in UACR levels as compared to non-Hispanics [23].

A post hoc analysis of the enrichment period of the SONAR trial reported that there was high variability in the albuminuria lowering effect and BNP altering of atrasentan between patients [27,31]. These differences between individual patients were attributed to pharmacokinetic and pharmacodynamics differences of plasma exposure to the drug. This finding is an important step towards defining individual patient treatments, developing therapeutic windows, and determining how the same drug will affect different patients. This inter-individual variability is important for future purposes, as we can likely alter doses according to specific patients. For example, since Black patients are less vulnerable to the effects of atrasentan, they can receive higher doses if required [31].

It was found that those with higher baseline albuminuria and lower baseline BP would better benefit from the albuminuria reducing the effect of atrasentan [17]. On the other hand, higher doses and lower GFR were associated with more fluid retention [24,31]. This counters a post hoc study of the ASCEND trial, which demonstrated that in patients who developed CHF, those in the avosentan group had a higher eGFR than those in the control group [24].

Pena et al. [26] studied the effect of ET receptor antagonists on urinary metabolites known to reflect mitochondrial protein function. The increased levels of metabolites found directly correlated with changes in eGFR [26]. Since lower metabolite concentrations reflect reduced mitochondrial content and renal function, it can be supposed that ET receptor antagonists stabilize aspects of renal mitochondrial function in DKD since ET-1 is known to play a role in regulating mitochondrial biogenesis [26]. This provides a new hypothesis for a beneficial reno-protective effect of atrasentan; however, the metabolites do not correlate with changes in albuminuria.

ET Receptor Antagonists in Combination With Other Drugs:

Patients receiving maximum doses of RAAS inhibitors had a similar response to those receiving other doses, which might imply that patients do not need to be on maximum therapy after all [23]. This can be beneficial for those who experience side effects of RAAS inhibitors, such as hyperkalemia. Nevertheless, there is still evidence that ACE inhibitors/ARBs and ET receptor antagonists work synergistically. Another possible synergistic relationship may exist between ET receptor antagonists and the SGLT2 inhibitor canagliflozin, which can reduce glomerular hyperfiltration and provide anti-inflammatory effects [35]. Combining canagliflozin and atrasentan may reduce the fluid retention of atrasentan while also providing an albuminuria decreasing effect [35].

ET Receptor Antagonists in the Future:

Other factors may affect the efficacy of ET receptor antagonists that are yet to be explored. As Yuan et al. [32] pointed out, patients with diabetic nephropathy are mostly above 60 years and are already prone to developing edema and hypotension. Perhaps early intervention with ET receptor antagonists can be beneficial in preventing the progression of diabetic kidney disease while also having a less likely chance of developing adverse effects. There is also a need to study the effects of bosentan and atrasentan further and compare them to the effects of atrasentan in a single trial.

Schievink et al. [25] suggested that exposing all patients to atrasentan may be harmful, and therefore an 'enrichment' period should be used to protect patients. Heerspink et al.'s [30] trial was the first to include an enrichment period that would enable them to select those patients who would most likely benefit from treatment to avoid excessive adverse events. Even with the cautionary strategies employed in this trial, which included the use of diuretics, hospital admission for congestive heart failure was still higher among responders who received atrasentan than in the control group. Therefore, future trials may need to develop newer ways to categorize patients to minimize adverse events. This also suggests that including only participants with an albuminuric response may not adequately identify those most likely to benefit [36].

Limitations:

Some of the trials included in this systematic review had few participants, which means that the results could largely depend on the larger-scale trials. The duration of treatment and follow-up periods also varied in trials and could have affected outcomes and adverse events reporting. The research on ET receptor antagonists is still limited as more studies have been conducted on atrasentan than avosentan or bosentan. Early termination of the ASCEND and SONAR trials leading to shortening of follow-up could have also provided incomplete results. Trials that excluded patients for whom the drug may not be beneficial may have been favorable in terms of preventing adverse effects; however, it is uncertain whether the approach to omit those patients was correct.

## Conclusions

Endothelin receptor antagonists have shown high efficacy in reducing albuminuria in patients with diabetic kidney disease. More specifically, there is greater evidence of atrasentan demonstrating enhanced effects on albuminuria reduction than avosentan and bosentan, with lower doses also causing minimal adverse events. In addition to this favorable effect on albuminuria, ET receptor antagonists also play a role in blood pressure control and improve lipid profiles. This systematic review helps in establishing a beneficial role of the ET receptor antagonists. However, to further prove the effectiveness of these drugs, more research is still required. This review highlights the inter-individual variations in drug effects based on various factors, such as demographic and lab values. These differentiating factors may be used to conduct additional studies using specifically designed methods to select the most appropriate participants and doses of the drugs. Planning in these regards is necessary in order to achieve better results while also preventing specific adverse effects. 
